# Ten simple rules for leading a many-author non-empirical paper

**DOI:** 10.1371/journal.pcbi.1013283

**Published:** 2025-08-14

**Authors:** Tracey L. Weissgerber, Natascha Drude, Rima-Maria Rahal, Friederike E. Kohrs

**Affiliations:** 1 Berlin Institute of Health at Charité – Universitätsmedizin, QUEST Center for Responsible Research, Berlin, Germany; 2 CNC-UC, Center for Neuroscience and Cell Biology, University of Coimbra, Coimbra, Portugal; 3 CIBB, Center for Innovative Biomedicine and Biotechnology, University of Coimbra, Coimbra, Portugal; 4 Max Planck Institute for Research on Collective Goods, Bonn, Germany; 5 Vienna University of Economics and Business, Vienna, Austria; Dassault Systemes BIOVIA, UNITED STATES OF AMERICA

## Abstract

Many-author non-empirical papers include recommendations or consensus statements, catalogs of ideas, roadmaps for future research, calls to action, or “how to” articles. These papers have great potential to change the conversation or address unmet needs within research communities. Large, diverse authorship teams can create valuable resources that no individual co-author could create independently. Achieving these goals, however, requires a very different approach than researchers typically use to prepare papers with fewer authors. In the process we describe, a small team of lead writers typically leads the content generation and writing processes. Many co-authors collaborate to create content and provide feedback throughout the writing process. Lead writers face many challenges, including defining the content and structure of the paper, coordinating complex logistics, preparing themselves and co-authors for a unique writing experience, and managing high-volume feedback. Here, we outline ten simple rules for leading a many-author non-empirical paper. These rules guide readers through the content generation and writing processes and highlight practical solutions to common challenges. While these rules were developed by preparing non-empirical papers with at least 30 authors, some rules may apply to research papers or non-empirical papers with fewer authors. Lead writers can also use our companion paper, which shares ten simple rules for being a co-author on a many-author non-empirical paper, to prepare co-authors for an efficient and effective collaborative process.

## Introduction

**Fig 1:**
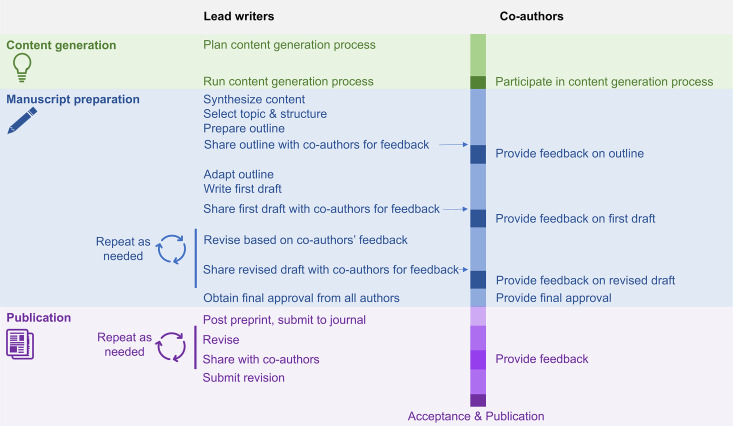
The process of preparing a many-author non-empirical paper. The infographic illustrates the activities of lead writers and co-authors during the three phases of completing a many-author non-empirical paper – content generation, manuscript preparation, and publication. Lead writers are typically highly involved in organizing the content generation process and writing the paper, while co-authors participate in content generation and provide feedback on the manuscript at specific points in the process. The general process shown here may be adapted based on the needs of the project or project team.

Many-author non-empirical papers bring together large teams of experts to explore topics that are important to a research field, to the entire research community, or to the public. These papers typically create a new resource or expand the research community’s understanding by building a collaborative synthesis that the individual authors could not have developed independently. Many-author non-empirical papers do not simply combine sections of text written by individuals, describing their own work, into a single paper. The highly interactive process typically includes an intense content generation phase, followed by a writing and consolidation phase with multiple feedback rounds (Fig 1). While there are no defined criteria for what constitutes a many-author paper, the rules described in this article were developed while writing papers with at least 30 highly engaged authors. Some rules, such as rules 2, 5, 6, 8, and 9, may be useful for papers with fewer authors or for many-author research papers. Notably, a previous ten simple rules article provides advice for conducting and writing a multi-author research paper with at least three authors from two different institutions [1]. Strategies for collaboratively writing Massively Open Online Papers have also been shared [2]. In the present paper, non-empirical indicates that the paper is not original research. Possible formats of non-empirical papers include recommendations or consensus statements, catalogs of ideas, roadmaps for future research, calls to action, or “how to” articles.

Writing a many-author non-empirical paper is challenging for several reasons. First, deciding on content and structure can be quite difficult. When writing an original research paper, the content is the authors’ research, and papers follow the Introduction–Methods–Results–Discussion, or IMRD, structure. In contrast, the authors of a many-author non-empirical paper could potentially write many different papers, using many different structures. After deciding which paper to write and what content to prioritize, lead writers must design a structure to clearly convey the desired message(s). Second, researchers often have limited experience writing or critiquing non-empirical papers that aren’t narrative reviews, editorials, or commentaries. Many co-authors may be contributing to a recommendation or consensus statement, roadmap for future research, call to action, or a “how to” article for the first time. This “learning while doing” approach can affect feedback quality. Third, all contributors must adapt to accommodate high-volume feedback, while balancing many diverse perspectives.

Despite these challenges, many-author non-empirical papers have important advantages for the scientific field and benefits for leads and co-authors as well. Large teams of very diverse experts lead to more comprehensive and nuanced perspectives than one might expect from a smaller authorship team. The resulting papers resonate with many different audiences, enhancing dissemination and applicability. Many-author non-empirical papers can bring together diverse perspectives, highlighting points of consensus and dissents in a scientific field. For leads and co-authors, the networks and new connections formed while preparing the paper can launch lasting collaborations and future actions as well as increase visibility and reach. Authors of a many-author paper can quickly get an overview of different perspectives and positionalities in the field. All of these factors can amplify the impact of the published work and be especially beneficial to early career researchers (ECRs).

Many-author non-empirical papers may be led by a small group of “lead writers” who design and run the content generation process and do most of the writing and consolidation. The authorship team also includes many co-authors, who participate in the content generation and provide feedback on the paper outline and drafts at defined timepoints throughout the process. The experiences and responsibilities of lead writers and co-authors are very different (Fig 1). Leading a many-author non-empirical paper is an intensive time commitment. Responsibilities include managing the complex logistics of content generation and manuscript preparation, making decisions about the scope, content, and structure of the paper, incorporating feedback from many co-authors and resolving disagreements. Behind the scenes, lead writers make many decisions and handle situations that are not apparent to co-authors.

This paper provides ten simple rules for leading a many-author non-empirical paper (Table 1). These rules can be used by experienced lead writers who would like to refine their content generation and writing strategies, as well as by ECRs who aim to lead larger collaborative projects and would like to gain additional skills. We encourage readers to consult the companion article, “Ten simple rules for being a co-author on a many-author non-empirical paper”, for advice on how co-authors can contribute effectively [3]. While some of the rules presented in these papers are specific to non-empirical papers, others, including rules 2, 5, 6, 8, and 9, may also apply to research papers.

**Table 1:** Ten simple rules for leading a many-author non-empirical paper.

**Rule 1**	Maximize collaborative synthesis when generating content
**Rule 2**	Choose motivated contributors who have varied expertise
**Rule 3**	Select a structure and writing style that aligns with your goals
**Rule 4**	You can’t make everyone completely happy, including yourself. Accept this before you start
**Rule 5**	Organize the writing process
**Rule 6**	Manage the accordion effect
**Rule 7**	Rethink everything you know about handling peer comments
**Rule 8**	Build consensus. Prevent or de-escalate conflicts.
**Rule 9**	Handle direct feedback from passionate individuals strategically
**Rule 10**	Anticipate challenges during the publications process

### Rule 1: Maximize collaborative synthesis when generating content

Use a content generation process that maximizes interaction and collaborative synthesis while breaking down hierarchical structures (see Box 1 for examples). The goal is to build on participants’ collective expertise to capture broad knowledge and create something that individual participants could not develop on their own. Embrace formats where participants share and learn through conversations, rather than presentations. Use facilitation techniques that allow conversations to flow among all participants, instead of through the moderator or the organizing team. Ask broad questions that engage everyone. Connect participants with specific knowledge to others with similar interests to motivate them to share their expertise. Add breadth and nuance to the discussion by encouraging participants to approach the topic from many different perspectives. Examine connections among ideas, unexplored areas, opportunities for advancement, and major challenges. Note points of consensus, while exploring the reasons for disagreements.

Avoid traditional symposium or conference formats, where selected speakers give talks; then answer a few questions. These formats hinder collaborative synthesis by prioritizing one-way communication, where information flows from the speaker to a passive audience. Participants have limited opportunities to critically evaluate others’ work or connect ideas. Furthermore, talks often share published information, and there may be systematic bias in participation. Speakers are typically well-known researchers or others with close connections to the event organizers, limiting participation of ECRs, researchers from underrepresented groups, or those who are outside the organizers’ network. Speakers and senior researchers often dominate discussions.

Organizers and participants create an inclusive space, where everyone can safely contribute, to facilitate collaborative synthesis. Due to the non-hierarchical and less structured nature of these non-traditional activities (Box 1), many participants are often ECRs. Compared to senior scientists, ECRs are more diverse and more likely to come from historically marginalized groups [4]. Content generation event organizers should work to maintain an inclusive space by using tools such as codes of conduct, and encouraging participants to be respectful of differing experiences, ideas, and perspectives, and leave room for intellectual disagreement, and embodying these behaviors themselves. This is particularly important for participants in positions of power, due to seniority, reputation, involvement in organizing and running the event, or other factors. Organizers can use group-based participation formats. Instead of asking individuals to speak up spontaneously, they invite input through small-group discussions, written reflections, or shared feedback forms. This lowers the barrier to entry and helps ECRs feel supported. Organizers can also ask participants to designate “spokespersons”, who are assigned or volunteer to act as a voice for ECRs during meetings or discussions. This ensures ECRs' ideas are heard even if individual ECRs are not comfortable voicing their own perspectives. Alternatively, organizers can use mentorship pairing or peer-support groups to formally or informally pair ECRs with a more experienced researcher who can support ECRs in building confidence, offering guidance, and demystifying group dynamics. Organizers can also rotate facilitation and feedback roles, by giving ECRs structured opportunities to lead an activity within the event or present informal updates. Rotating roles encourages ownership and balanced contributions. Finally, organizers can check in with ECRs individually, as some ECRs may feel more comfortable sharing feedback or ideas in one-on-one discussions. The approaches and behaviors used to create a safe and inclusive space should extend throughout the project, including the content generation, writing, revision, and publication phases.

In papers written through virtual brainstorming events organized by the authors, early and mid-career researchers have made up the majority of both participants and the lead writing teams, and benefits for them have been described elsewhere in this paper. Actively encouraging the participation of ECRs is also beneficial for content generation as ECRs are the largest and most diverse group of researchers [4,5] and bring in a wealth of expertise on various topics addressed in non-empirical papers.

**Box 1. Content generation strategies**

A few examples of content generation strategies that the authors have successfully used to write many-author non-empirical papers are listed below; however, this is not an exhaustive list. Lead writers can explore other formats, such as BarCamps [6] or World Cafés [7], or adapt existing formats to suit their needs. Regardless of the format chosen, lead writers should have a clear plan to focus conversations on topics that are especially important for the creation of the resulting paper.

**Unconferences**

Unconferences, or unconventional conferences, seek to maximize the discussions and networking that occur during coffee breaks at traditional conferences. There are many different unconference formats, for in-person and virtual meetings. Examples include structured conversations (see the “Participant led workshops at the Future of Research Symposium” section of [8]) and virtual brainstorming events [9,10]. Virtual brainstorming events bring together researchers and other stakeholders for two days of asynchronous discussion on a specified topic [9,10]. The two-day events include virtual networking events, where participants get to know each other, webinars, where participants give lightning talks, virtual synchronous meetings for small group discussions on important topics, and lively asynchronous discussions on an online discussion board (e.g., Slack). Other ideas for starting focused conversations on topics relevant to the theme of an intended paper might include asking participants to discuss controversial statements or PechaKucha talks [11], comprised of 20 slides each shared for 20 s, where participants share a specific idea or experience. The unconference format was developed for global teams (e.g., [5,12]); however, it can also be used nationally (e.g., [13]) or locally.

**Meetings**

Lead writers may organize a series of in-person or virtual meetings, where contributors share expertise and experiences. A moderator guides the discussion. Small groups of 4–6 people allow everyone to contribute. Organizers may either hold a series of small group meetings, or use breakout groups or rooms to facilitate small-group discussions, before bringing everyone together to share insights gained from each group.

**Intensive workshops**

Organizers establish guiding questions or themes independently, or in consultation with workshop participants. The workshop itself may combine small group discussions, where participants explore particular questions in depth, and large group discussions, to synthesize and share information among all participants. Conversations are documented by notetakers, or by using interactive tools or online whiteboards to complement brainstorming and discussion exercises.

**Writeathons and writing sprints**

Lead writers may organize one or more collaborative, time-limited events to bring together various contributors, who work on achieving predefined content generation goals. Outputs for a session may include drafts of chapters or paper sections, or illustration mock-ups. Writeathons or writing sprints can facilitate rapid collaboration and content generation.

### Rule 2: Choose motivated contributors who have varied expertise

Many-author papers are especially powerful when they bring together individuals who have diverse perspectives, due to differences in expertise, field, career stage, country, identity, or other factors. The co-authorship team may also include individuals from different stakeholder groups (e.g., researchers, patients, funders, publishers, and editors). Look for experts from many different fields who can offer different perspectives on the topic. Go beyond your existing network. Do research to identify a global team of people who are actively working on the topic. Content generation events typically attract people who are there to actively contribute, as well as people who are there to learn. The people who are there to contribute drive the conversation, so it’s essential to have a large group of motivated contributors. Ask senior people who might not have time to participate to recommend one or more early career colleagues. Invite people in non-research roles who have relevant expertise, such as librarians, administrators, and support staff. Inviting representatives of other stakeholder groups, such as funders, publishers, patient organizations, or non-governmental organizations, may further strengthen the discussion and expand the pool of knowledge.

Selecting lead writers is also crucial, as lead writers drive the project. The writing team should include skilled writers who are adept in building consensus without insisting on their own convictions, managing conflict, and synthesizing large quantities of detailed information to identify broad themes and unique opportunities. Include someone with strong organizational and communication skills, who can update co-authors regularly and address personal concerns. The lead writers should form a cohesive, collaborative team which is open to leads’ and to co-authors’ differing points of view, leaving space for intellectual disagreement. Leading a many-author non-empirical paper is very time consuming, so it’s important to choose highly motivated people who can dedicate the time needed. You are probably underestimating the amount of time that this process will take. Time requirements increase with the number and engagement level of co-authors, and the volume of information shared during the content generation phase.

Early and mid-career researchers have been involved as lead writers in many of the papers that we have completed, allowing them to gain practical experiences and refine skills, building networks and collaboration opportunities while increasing their visibility. A lead writing team fully consisting of ECRs may benefit from collaborating with a more senior colleague who has experience in leading a many-author paper and can provide mentoring and guidance.

### Rule 3: Select a structure and writing style that aligns with your goals

Before starting the writing process, be specific about the goal of your paper and the gap that you aim to address. If the paper’s goals and structure are not clearly defined before content generation, topic selection is both a challenge and an opportunity. Even when the goals and structure are pre-defined, the focus may shift as new ideas are introduced. Successful content generation events generate a lot of information. After a virtual brainstorming event, for example, there may be 5–10 papers that one could write. Don’t attempt to summarize everything that was discussed. Table 2 outlines different formats of non-empirical papers. Select a topic and format that fills a gap by meeting a community need. For example, you might prepare a recommendation or consensus statement if different perspectives among researchers are hindering advancement, or you might create a community resource or “how to” guide to eliminate barriers and accelerate progress. Ensure that the paper that you will create is needed and doesn’t duplicate existing resources. Prioritize broad topics that build on the knowledge of many contributors, over narrow topics that could be addressed by a few people with specific expertise. When defining your goals, pay special attention to underdeveloped areas and unique ideas that should be explored further.

**Table 2:** Types of many-author non-empirical papers.

Paper type	Goals and examples
Recommendations or consensus statements	Recommendations for achieving a particular goal [5]^*^
Catalog of ideas	Shares many ideas or approaches for achieving a particular goal, and may also briefly describe examples or cite resources for implementing each idea [13]
Call to action	Encourages stakeholders to take action on a specific topic, and outlines why this action is needed [14]
Roadmap for future research	Identifies key unsolved problems that are crucial for the advancement of a particular field
“How to” papers	Shares expert knowledge on how to do something, by providing practical input and examples [12]
Community resource	Creates a resource that others can use and build upon
Combination papers	Combines two or more of the categories above (e.g., [15] is a recommendation, roadmap, and “how to” paper)

* Recommendations presented in non-empirical papers are generally developed through expert consensus, rather than systematic approaches, such as a Delphi processes [16]. While there are many examples of these types of works, we have only cited works in which the authors of these ten simple rules papers participated. Other groups may have different content generation and writing processes, and we do not want to imply that they used the processes outlined in this paper.

Content generation events often include lengthy discussions of popular topics that many people are working on. These discussions typically reiterate information that has been shared elsewhere. Look for “hidden gems” and focus on what your group can offer that no one else can provide. Some of the most powerful ideas may have only been mentioned briefly during the content generation phase.

Consult other papers that used the format that you selected to explore possible structures, and adapt these structures as needed for your content. Test and refine the structure and content throughout the writing process to effectively convey the paper’s main message(s). Clearly define your target audience; then write for your target audience, not for yourself. The structure, content, wording, writing style, and visualizations will differ if you are addressing researchers in your field, researchers working in many different fields, or the public.

We advise against writing “symposium summary” papers that summarize event discussions, without presenting a collaborative synthesis. Symposium summary papers often jump from topic to topic, resulting in a disjointed paper with no cross-cutting theme or message. This effect may be especially pronounced if each speaker writes a section. Sections describing speakers’ talks can feel like advertisements if the authors don’t address limitations. Furthermore, sections may briefly summarize published work, without adding new information. Readers may have difficulty distinguishing between opinions and statements supported by evidence. Missing citations exacerbate this problem, and they are common in symposium summaries as talks may omit relevant citations. Empty sentences stating that a topic was discussed should be replaced with text describing the substance and relevance of the discussion. Authors can avoid some of these limitations with careful writing and extensive editing to harmonize the content, technical terms, language, and writing styles across sections. Symposium summaries, however, are most useful when they have another goal, such as sharing a roadmap for future research, or providing consensus recommendations.

Lead writers may want to adjust their writing style to suit the paper’s goals. “How to” papers, for example, are often written in an active voice. Calls to action may encourage specific stakeholders to carry out the actions described in the paper. Recommendations may provide customized recommendations and tailored wording for different stakeholder groups.

Co-authors on many-author papers will have diverse epistemic backgrounds, and terms and concepts may differ across fields. A mutual understanding and consensus of terms amongst co-authors and leads may be reached through iterative exchange during the revision process and can evolve organically without the leads providing strict definitions. Variations or dissents about terms or concepts can also be showcased in a manuscript to explain the reasons for the lack of consensus (see [17] for an example).

### Rule 4: You can’t make everyone completely happy, including yourself. Accept this before you start

When working with a few authors, the lead writers draft the paper, get feedback from co-authors, revise, and submit the paper once all co-authors approve the final version. This process is more complex with a large author team. The lead writers draft the paper and revise in accordance with each other’s feedback. The lead writers then share the paper with at least 30 highly engaged co-authors, who provide very detailed feedback about everything that they dislike or think could be improved. Co-authors don’t always agree. The lead writers’ mission is to find a path forward and build consensus (see the section in Rule 7 on identifying opportunities for transformative improvement and Box 2). Sometimes, that consensus may not fully align with the leads’ own views.

The limited feedback that lead writers receive on non-empirical papers with few authors preserves the illusion that the paper is excellent – only a few problems were identified, and they were fixed. In contrast, lead writers in many-author non-empirical papers may receive hundreds of comments during one feedback round. The revisions made to incorporate this extensive feedback ideally lead to a more comprehensive and nuanced paper that will resonate with a wider range of audiences. This process eliminates the perception that the paper is perfect, as some problems can’t be solved. Leads have a very detailed understanding of the things that people may dislike, misinterpret, or don’t understand about the paper. They may know approximately what proportion of people fall into each category, and how these issues vary across different audiences. Lead writers also receive private emails from passionate co-authors who are dissatisfied with particular aspects of the paper (see Rule 9). Compromise is essential, as adding language that satisfies passionate individuals may trigger backlash from other co-authors. Leads have to simultaneously guide and follow the consensus building process, displaying intellectual humility and remaining open to compromise even if the final message does not fully reflect their own perspectives.

Lead writers know many of the papers’ unfixable flaws before journal peer review. Despite this, papers are typically stronger because the team fixed many problems that would not have been identified without a large, diverse authorship team. You can’t make everyone happy, including yourself, but you can bring people together to write a stronger paper through collaborative synthesis.

### Rule 5: Organize the writing process

Good organization improves efficiency. In addition to organizing the content generation activities (Rule 1), lead writers need to structure the writing process. Decide at which stages in the process you will request feedback. We suggest requesting feedback on the manuscript outline, followed by a first draft, and one or two revised drafts. At each stage, decide where you will share materials so that co-authors can comment. Use an accessible platform with collaborative editing software, such as Google docs or Sharepoint, that allows co-authors to see each other’s comments. Clearly specify when materials open for comments, and close for revision. Some co-authors will provide feedback soon after receiving the materials. Others will enter comments when you send a reminder, a few days before the feedback period ends. A small proportion of authors will email you on the last day to ask for an extension. This happens on every paper, no matter how long the comment period is, so plan accordingly. If you plan to close the commenting period and start editing in four weeks, be strategic and tell authors that the commenting window is three weeks. You can then give people an extra week, without disrupting your editing schedule.

The commenting period should be a minimum of two weeks, but may be extended to 4–5 weeks during vacation periods. Schedule the comment period at a time when you are busy to avoid the temptation to address comments as they come in. Comments are most useful in aggregate (see Rule 7), so wait until all authors have reviewed the draft. Ask authors to add their name, affiliation, and ORCID, and record any conflicts of interest on the first draft, after they comment. Request this information in a clearly visible space, at the top of the draft, rather than collecting this information in a separate file.

Each time that you share materials, include tips for co-authors on how to provide effective feedback. This is critically important, as most co-authors won’t have worked on a many-author non-empirical paper before. Post this information at the beginning of the file containing the outline or draft. Sample tips are shared on the Open Science Framework [18]. When sharing a revised draft, include a summary of revisions. Explain (1) the major changes that you made, and the reasons why you made them, (2) why you didn’t implement commonly suggested changes, (3) what type of feedback is most helpful at this phase, and (4) constraints that affect how co-authors should approach the next revision (e.g., “we are close to the word limit”). Request input on where to post a preprint or submit the paper for publication, and ask authors to notify you if their contact information changes before the paper is published.

Be clear about any plans for authorship and communicate them to co-authors from the beginning, including criteria for being an author. While quantifying exact contributions with many co-authors is difficult, leads can specify the timepoints in the content generation and writing process when co-author input will be expected, and the level of input needed. Contributions at different timepoints in the content generation and writing process can affect the roles of co-authors accredited by the CRediT taxonomy (https://credit.niso.org/). For most co-authors, participating in the content generation phase is the intellectual contribution to authorship, fulfilling the “Investigation” role in CRediT. Co-authors will also typically fulfill the “Writing – revising and editing” CRediT role by providing feedback on the outline and/or manuscript drafts. As outlined in the companion paper for co-authors [3], author order may not be particularly meaningful on a many-author paper. Lead writers will often be listed in the beginning and end positions, with co-authors listed as middle authors. The order of middle authors may be randomly assigned or alphabetical. Alternatively, some groups may use consortium or group authorships [19,20]. Lead writers are named in the authorship list, and co-authors are included in the writing consortium.

### Rule 6: Manage the accordion effect

*(Irrelevant note: TLW wrote this section while sitting in a park next to a café that was playing accordion music. She did not enjoy it and doesn’t think that this improved the content.)*

**Fig 2:**
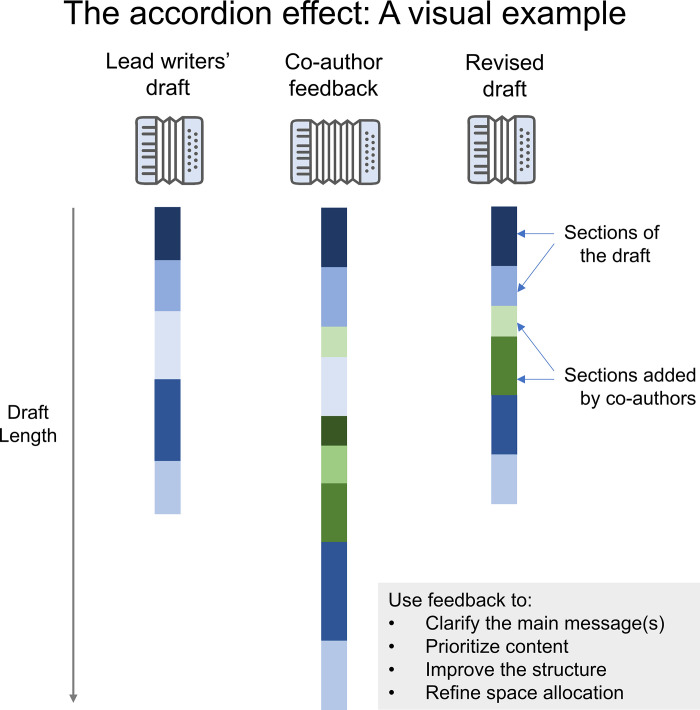
A visual example of the accordion effect. Colored bars represent different sections of the manuscript, and the bar height illustrates section length. Blue sections were originally drafted by the leads, and green sections were added by co-authors. The number and length of sections increases after co-authors comment, and the lead writers must make the paper more concise while incorporating feedback received during revision.

Many-author papers typically become much longer each time that they are opened for comments, and lead writers must make them more concise each time that they are closed for editing. We call this phenomenon “the accordion effect.” This occurs because most co-authors will add things, but very few will delete text or comment on things that could be removed. Fig 2 illustrates the accordion effect during one feedback cycle of a hypothetical paper. Colored blocks represent different sections of the paper. The lead writers shared a draft with five sections. After the comment period, each of these sections is longer and several short sections have been added. After the lead writers finish incorporating feedback, the revised manuscript has six sections, and each section has been shortened. Two of these sections were added by co-authors. Use feedback to clarify the main message(s) of the manuscript, prioritize content, improve the structure, and refine space allocation.

There are several additional things that you can do to manage the accordion effect. Clearly define the manuscript’s scope, goals, and audience, and share this information with co-authors. This helps co-authors to determine whether content that they might like to propose is relevant. When soliciting feedback, state the target journal’s word limit and the draft word count at the top of the instructions for authors, in large, brightly-colored font. When the draft is close to the word limit, remind authors that any additions must be balanced by deletions, so newly proposed content must be more important to the main message(s) than existing content. Encourage participants to post comments if they would like to add something. Lead writers can then contact co-authors to request text for additions that they want to include. When requesting text, specify the desired length and content. Create an “idea parking lot” at the end of the draft where co-authors can share ideas that may not be within scope for the current draft, but could be explored in future papers. When incorporating feedback, remove content that isn’t essential to the main message(s) of the paper. Co-authors frequently add text on popular topics, and this text often duplicates existing resources. Replace text that isn’t essential to the main message(s) of the paper with a sentence encouraging readers who want more detail to consult comprehensive resources. Ask co-authors to suggest references. Encourage co-authors who are passionate about topics that are out of scope to collaborate on a separate paper.

### Rule 7: Rethink everything you know about handling peer comments

Researchers who write papers with fewer co-authors typically address comments from co-authors or peer reviewers one at a time. This approach works when you have a modest number of comments that focus on isolated issues. Many-author papers, however, can receive hundreds of interconnected comments and edits during the first revision. Some of these comments will directly conflict, and co-authors may debate the merits of suggested changes. This extensive feedback is a second content generation phase, which can lead to transformative improvements of the draft. Lead writers need to approach comments completely differently to take advantage of this opportunity. Once you’ve learned the strategies described below, you’ll find that many of these techniques are also useful for papers with fewer authors.

#### Develop organizational strategies for managing high-volume feedback:

Leave the original file unchanged to preserve all feedback. Edit in an offline copy of the commented file. Revising the draft is a complex, iterative process, so working offline allows lead writers to experiment with revisions, without inadvertently notifying co-authors each time a comment is accepted or rejected. Don’t attempt to respond to every comment. Maintain a separate file, where you describe major changes that you made to the draft, and common requests that were not implemented. In each case, describe the rationale for the lead writers’ decision. You’ll email this information to co-authors with the revised draft (see [18] for examples).

#### Identify opportunities for transformative improvements:

Making decisions about each comment individually is neither feasible, nor productive, with hundreds of interrelated comments. High-volume feedback is most useful in aggregate. Start by looking for common themes, or sets of related comments. This process is a bit like reconstructing artifacts from fragments found on an archeological dig. Some fragments don’t fit with anything else, whereas others fit together to form larger objects. Pieces from the same object may be clustered close together, or spread far apart. Objects won’t be complete, and you may not know what the object looks like until you find enough pieces that fit together. Some objects are more valuable than others.

The many comments provided by co-authors offer crucial insights to help you improve the paper, build consensus, and de-escalate conflicts. Focus on understanding the problems with the draft before you develop solutions. Some comments will provide a clear and accurate assessment of the problem. In many cases, however, you will need to combine comments from different co-authors to fully understand the problem. Researchers are very experienced in writing and critiquing original research papers, so their assessments of what is wrong and how to fix it are generally reliable. In contrast, this may be the first time that many co-authors are contributing to a guideline or recommendation, catalog of ideas, research plan, call to action, or “how to” paper. Co-authors may have difficulty identifying why something isn’t working, or how to fix it. In addition, co-authors may interpret the message(s) of the paper differently, depending on their expertise. Understanding how co-authors from different backgrounds interpret the paper will help you to refine the draft so that it resonates with many different audiences.

Look through all the comments to identify recurring themes, including suggested additions and things that co-authors found misleading or wanted to improve. Also look for places where co-authors misinterpreted your intended meaning, or areas where co-authors understood terms or concepts differently. Sometimes comments in completely different sections of the paper can provide insight into a single problem (Box 2). Identify common causes that underlie disagreements throughout the draft. This might include fundamental differences in the values, goals, approaches, or language used by different groups of co-authors. Identifying and explaining these underlying sources of disagreement can provide new insight for authors and readers, and prevent further conflicts.

Once you understand the major problems, think creatively about solutions. Look for solutions that simultaneously address all related problems. This often involves finding a third way (see Rule 9).

#### Prioritize changes:

Address groups of comments that require major changes to the structure and content of the manuscript first. Don’t waste time on minor edits to sections that may be cut. After finalizing the major changes, address minor comments in sections of the manuscript that have not changed substantially.

Structure is a common source of disagreements in many-author non-empirical papers. Sometimes the only way to determine whether a proposed structure will work is to try it. A proposed structure may sound promising; however, obvious flaws may emerge once you start writing. In cases where restructuring can be done quickly, compare both to determine which structure most effectively conveys the main message(s).

#### Provide co-authors with a summary of decisions and changes:

When you’ve finished revising, finalize the statement of major changes and common requests that were not implemented. Share this information with co-authors along with the next version of the draft.

**Box 2. A case study on the value of aggregated comments and finding a third way**

**The comments**

In a recent manuscript exploring how publishers could engage early career researchers in improving the future of scholarly communication [21], the lead writers observed disagreements throughout the manuscript over the magnitude of the problems with the scholarly publishing system, and the value authors placed on different solutions. We present a few representative examples below. Wording was changed to convey the main message to readers who have not read the preprint.

- The first paragraph of the introduction does not go far enough in emphasizing how broken and inequitable the publishing system is.
- The sentences added to the first paragraph of the introduction describing the problems with the publishing system are unnecessarily inflammatory and may offend those in publishing who have been working to improve the system for years. Some authors may not understand how difficult and lengthy a process it is to change this very complex system.
- The figure undervalues the important impact that early career researchers can have on improving scholarly publishing by acting as peer reviewers. As shown, peer review appears to be much less important than other activities, like serving on advisory boards, underselling its importance.
- We should discuss why early career researchers should invest time in improving publishing. What is in it for them? They are facing many obstacles in developing their own careers and have few resources. Why should they devote unpaid time to fix a broken system that undervalues them?
- Proposed strategies to involve early career researchers to improve the publishing system should be separated between those for developed and for developing or embargoed countries. Issues within the publishing system, such as article processing charges, can impact researchers disproportionally.
- The conclusion waters down the main message of the paper and doesn’t go far enough in emphasizing that publishers need to compensate early career researchers for the time that they invest in efforts to improve publishing.

**The problem**

Taken together (see Rule 7), these comments indicated that co-authors had different goals and were using fundamentally different approaches to improve scholarly publishing. Some co-authors wanted to improve the performance of editors and peer reviewers within the existing publishing system. These co-authors commented that the manuscript didn’t adequately acknowledge the value of these activities. Other co-authors wanted to improve or reform the publishing system itself, either through iterative (evolutionary) or transformative (revolutionary) change. These co-authors felt that the manuscript did not go far enough in critiquing the current publishing system. Some co-authors added harsher critiques and calls for transformative change, which raised concerns among more moderate co-authors who wanted to improve the performance of individuals within the current system.

**The solution**

**Fig 3:**
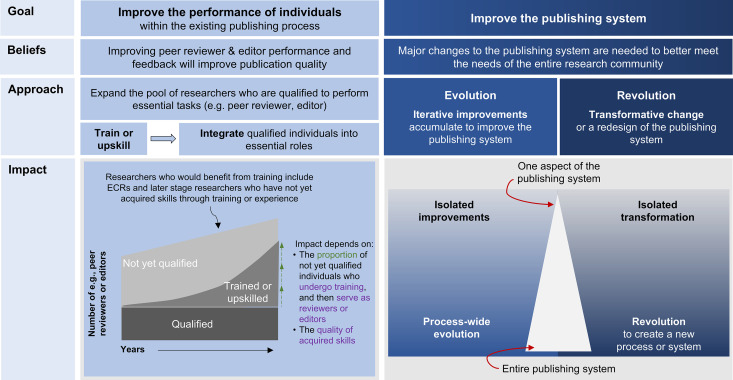
Approaches to improve scholarly publishing. The figure highlights differences in belief, approach, and the potential impact of approaches that seek to improve the performance of individuals within the existing publishing process, vs. those that seek to improve the entire scholarly publishing system through evolutionary or revolutionary approaches. Activities that focus on improving the performance of individuals typically seek to improve the quality of reviewer and editor feedback. Reproduced from [21].

The lead writers decided that it was not possible to convince everyone to embrace the same approach. Furthermore, doing so would have weakened the manuscript, as these tensions exist amongst researchers and publishing professionals who are working to improve scholarly publishing. Everyone working in this space should recognize these different approaches, and be prepared to collaborate with others who hold different views. In the revised draft, the lead writers found a third way (see Rule 8) by adding a new section and figure (Fig 3) to explain these different approaches. Acknowledging the diversity of perspectives provided essential, actionable information for readers, and eliminated the competing requests to adapt language in different parts of the manuscript to reflect one of these approaches.

### Rule 8: Build consensus. Prevent or de-escalate conflicts

Handling divergent or conflicting feedback is a common problem in many-author non-empirical papers. While diverse perspectives enrich the paper, they can also lead to conflicts. Researchers with different backgrounds may understand terms, concepts, or situations very differently.

There are many strategies that you can use to prevent or de-escalate conflicts. Explore the issue at different levels. Sometimes it’s possible to build consensus around higher level principles, even if authors have conflicting opinions about details. Distinguish between situations where you can build consensus, and situations where consensus is not possible or desirable (Box 2). In the latter case, acknowledge the controversy openly. Encourage both leads and co-authors to intentionally reflect on their own assumptions and viewpoints as this may help to identify sources of disagreements or conflicts [22]. Briefly outline the different positions, and rationale for each position, in the paper or through positionality statements in a supplementary file; then state the implications of this controversy for those working on this topic. This allows for points of dissent to remain visible and may encourage others to consider their positionality on the topic.

An alternative strategy is to find a third way. Look for creative solutions that reframe the discussion, or change the way that information is presented, in a way that works for most co-authors without undermining the goals of the paper. In rare cases, leads may consider setting up a meeting with co-authors involved to mediate a conflict or resolve serious misunderstandings due to language barriers or opposing viewpoints.

#### Practice defensive writing:

Defensive writing can prevent conflicts by preventing misunderstandings. Most readers are familiar with the term “defensive driving”, which refers to practices that drivers consciously use to prevent accidents. We use the term “defensive writing” to refer to conscious choices that writers make to improve clarity for readers from different backgrounds, and reduce the likelihood of misunderstandings. Pay careful attention to sources of misunderstanding, confusion, and controversy during content generation and while reviewing feedback on the draft. Work collaboratively with co-authors to refine the main message(s) so that they resonate with readers from many diverse backgrounds. As lead writers and co-authors gain experience working with diverse co-author teams, they will learn to identify or anticipate common sources of misunderstandings and controversy, and develop strategies for presenting content clearly.

#### Expect one or two inappropriately harsh comments:

When inappropriately harsh comments address aspects of the piece written by the lead writers, it’s typically best for the lead writers to wait for other co-authors to respond, instead of replying directly. Problematic comments will be obvious to most co-authors. One or more co-authors will usually explain why they disagree, suggest that the comment could have been more constructive, or explain why the language used is not appropriate even though they agree with the content of the comment. Leads are in a position of authority, and should not act too quickly as they might stifle the conversation. Waiting for other co-authors to respond avoids the perception that the lead writers are not open to criticism, while allowing co-authors to affirm the value of inclusive and respectful communication. If inappropriately harsh comments are directed towards other co-authors, it’s important for the leads to intervene quickly to de-escalate the situation and create a safe space for everyone.

### Rule 9: Handle direct feedback from passionate individuals strategically

Passionate individuals will often send you feedback directly. This includes emails from co-authors who want to privately offer sensitive feedback, or draw your attention to things that are important to them. Third parties may also contact you after you post the preprint. If the feedback is highly relevant, revise the paper and thank the person. Feedback from passionate individuals can cause problems if the proposed content is out of scope or other co-authors will disagree with the suggestions. Responding to emails from passionate individuals is time consuming, so start with prevention. When sharing tips on how to contribute effectively, encourage co-authors to post comments directly in the draft so that they are part of the collaborative conversation. This helps to build consensus and identify areas of controversy, instead of placing lead writers in the difficult position of making an executive decision about the feedback, or finding an anonymous way to share the feedback with the wider authorship team.

Sometimes the person contacting you will ask that their work be cited. If the resource isn’t relevant, or there is a better citation, be polite but firm. There are often many resources that could be cited to support a particular claim. Cite the resource(s) that will be most useful to readers. Resist pressure to cite papers of co-authors, especially when citations from other researchers are more suitable. When describing a phenomenon, provide one or two selected examples or citations instead of attempting to list all possible examples. Such lists are never comprehensive, quickly become outdated, and increase the likelihood that others will ask you to cite their work.

In other cases, a co-author or third party may have very strong opinions about an aspect of the paper. These opinions may not be relevant to the main message(s), or they may trigger backlash among other co-authors. Remind co-authors that this is a group writing process, and the goal is to build consensus, when possible, and accurately describe controversies. No one will be completely happy with everything (see Rule 4), including the lead writers. If necessary, set boundaries to prevent a single author from having veto power over any aspect of the paper. The writing process is collaborative, and each person can choose whether to be an author.

Occasionally, passionate individuals will contact you repeatedly with the same requests. Offer a clear, direct response; then quietly disengage if the conversation becomes circular.

### Rule 10: Anticipate challenges during the publications process

Many-author non-empirical papers can be challenging to publish, as the topic, goals, and structure of these papers often differ from the papers that journals typically handle. There are several things that you can do to mitigate these challenges. Obtaining final approval from all co-authors will take time. Plan for at least two weeks for all authors to review and sign off on the final version of the draft. Ask all authors to confirm that their affiliation, ORCID, and conflict of interest information is correct when reviewing the final draft, and to ensure that their conflict of interest statement complies with their institutional policies. When requesting approval on the final draft, also share your recommendations about where you might submit the paper and request feedback.

Post a preprint to create interest, using a preprint server that accepts non-empirical papers, such as OSF Preprints. Authors can share and solicit feedback on the paper, and you may receive invitations from journal editors. Look for journals that have published similar work. When possible, identify an editor who is supportive of the overall goals of the paper. Approach editors via pre-submission inquiries or informal emails to quickly find out whether the paper is within the journal’s scope. This avoids wasting authors’ and editors’ time with formal submissions if the paper is out of scope. Explain why you think that the paper would be of interest to the journal audience and how it might attract additional readers by appealing to others beyond the journal’s traditional audience.

As with authors, the editors and peer reviewers handling the paper may have limited experience assessing recommendations or consensus statements, catalogs of ideas, roadmaps for future research, calls to action, or “how to” articles. When possible, suggest reviewers who have experience in writing and leading these types of papers. Finding reviewers may be quite difficult if all experts in a particular topic area are co-authors on the paper. Use reviewer comments as a guide to what isn’t working about the paper; then be creative about generating solutions. Some reviewer or editor comments may mirror comments from co-authors during the writing process. In your rebuttal, explain the discussions that occurred during the writing process and the rationale for your decisions. Comments about manuscript structure are common, and may be driven by journal formatting requirements for specific article types. Work with the editors to identify the best solution for your paper. This may include changing article types, asking journals that don’t already have them to add features like text boxes, or other creative solutions.

Maintain contact with authors throughout the submission process. Update your mailing list and the affiliations in the manuscript when authors move. Send group updates and let authors know when you expect to contact them again (e.g., “The manuscript has been sent out for review. I will let you know when we receive a decision.”).

#### Limitations and scope

This paper highlights one strategy that we have successfully used for writing many-author non-empirical papers, however, other groups may use different processes. This may include workflows in which co-authors are separated into writing groups to prepare specific sections, receiving feedback from a team of lead writers. Alternatively, groups may use processes in which the authorship team expands or contracts as needed. Additional lead writers may be added to write sections in which they are particularly knowledgeable, or lead writers may need to step back to devote time to other projects. Regardless of which process is used, having a core team that drives the project forward is crucial; otherwise, the paper is unlikely to be completed due to diffusion of responsibility and competing priorities. When merging sections from different authors or teams, substantial editing and revising is often needed to ensure that the manuscript is cohesive and has a clear line of argumentation. Designating a small group who is responsible for this revision process may lead to more cohesive results.

## Conclusions

Many-author non-empirical papers offer a unique opportunity to create valuable resources that no individual co-author could create independently. These papers have great potential to change the conversation or fill unmet needs within research communities. Leading a many-author non-empirical paper, however, is a uniquely challenging experience. Essential skills include strong communication and organization, conflict resolution, defensive writing, and finding a third way. When selecting topics and content, lead writers should look for hidden gems and opportunities to create something that only a diverse authorship team can provide. Lead writers should be prepared for common challenges, like managing the accordion effect, disagreements among co-authors, and responding to feedback from passionate individuals. Embracing the chaos in a many-author paper means turning the diversity and complexity of contributions into a strength. Lead writers must encourage authors to bring unique perspectives by sharing their diverse expertise and creativity, while ensuring that the paper’s overall narrative remains cohesive. Leading a many-author non-empirical paper is a marathon, not a sprint. There will be periods of intense activity, followed by pauses when you are waiting for feedback or are busy with other projects. Pace yourself and stay focused on the end goal. Working collaboratively with other lead writers to support each other will help you maintain your motivation. Like many challenging skills, learning to lead a many-author non-empirical paper takes time and practice. Collaborating with others who are experienced can accelerate skill development. Every paper and authorship team is unique, but the first paper is typically the most challenging. Subsequent papers become easier as you expand your toolkit for handling common situations. Lead writers play a valuable role in facilitating and channeling the creativity of a large and diverse authorship team, and the research community needs more researchers who have this essential skill.
